# Ethidium hepta­fluoro­butyrate

**DOI:** 10.1107/S2414314622008847

**Published:** 2022-09-08

**Authors:** Runa Shimazaki, Masaaki Sadakiyo

**Affiliations:** a Department of Applied Chemistry, Faculty of Science Division I, Tokyo University of Science, 1-3 Kagurazaka, Shinjuku-ku, Tokyo, 162-8601, Japan; Zhejiang University (Yuquan Campus), China

**Keywords:** crystal structure, ethidium salt, hepta­fluoro­butyrate

## Abstract

In the title compound (systematic name: 3,8-diamino-5-ethyl-6-phenylphenanthridin-5-ium 2,2,3,3,4,4,4-heptafluorobutyrate), C_21_H_20_N_3_^+^·C_4_F_7_O_2_^−^, two ethidium ions, C_21_H_20_N_3_^+^ form a dimerized structure due to π–π inter­actions, even though they are positively charged. The hepta­fluoro­butyrate anions are connected to neighbouring cation dimers *via* hydrogen-bonding inter­actions, the hydrogen-bonding donor sites of the –NH_2_ groups of the ethidium ions connecting to the hydrogen-bonding acceptor sites of the –COO^−^ groups of the hepta­fluoro­butyrate anions.

## Abstract 2

The crystal structure of the title ethidium salt has been determined. The ethidium cations construct a dimerized structure due to π–π inter­actions.

## Graphical abstract

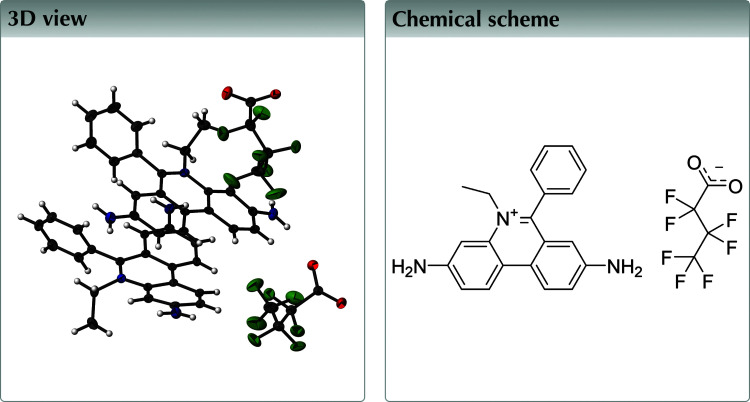

## Structure description

**Figure 1:**
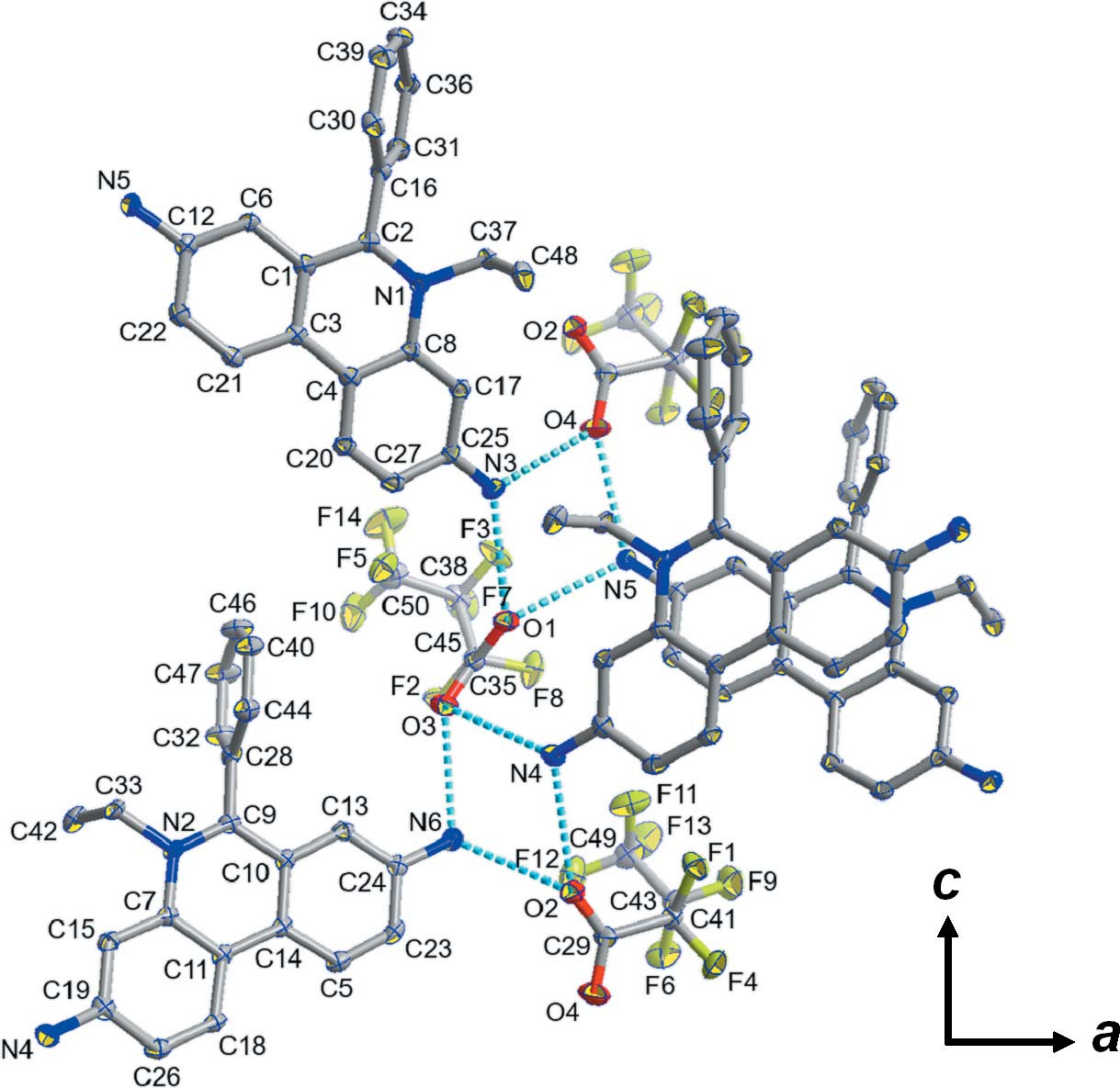
The crystal structure and hydrogen-bonding arrangements of the title compound with displacement ellipsoids drawn at the 50% probability level. Hydrogen atoms are omitted for clarity.

**Figure 2:**
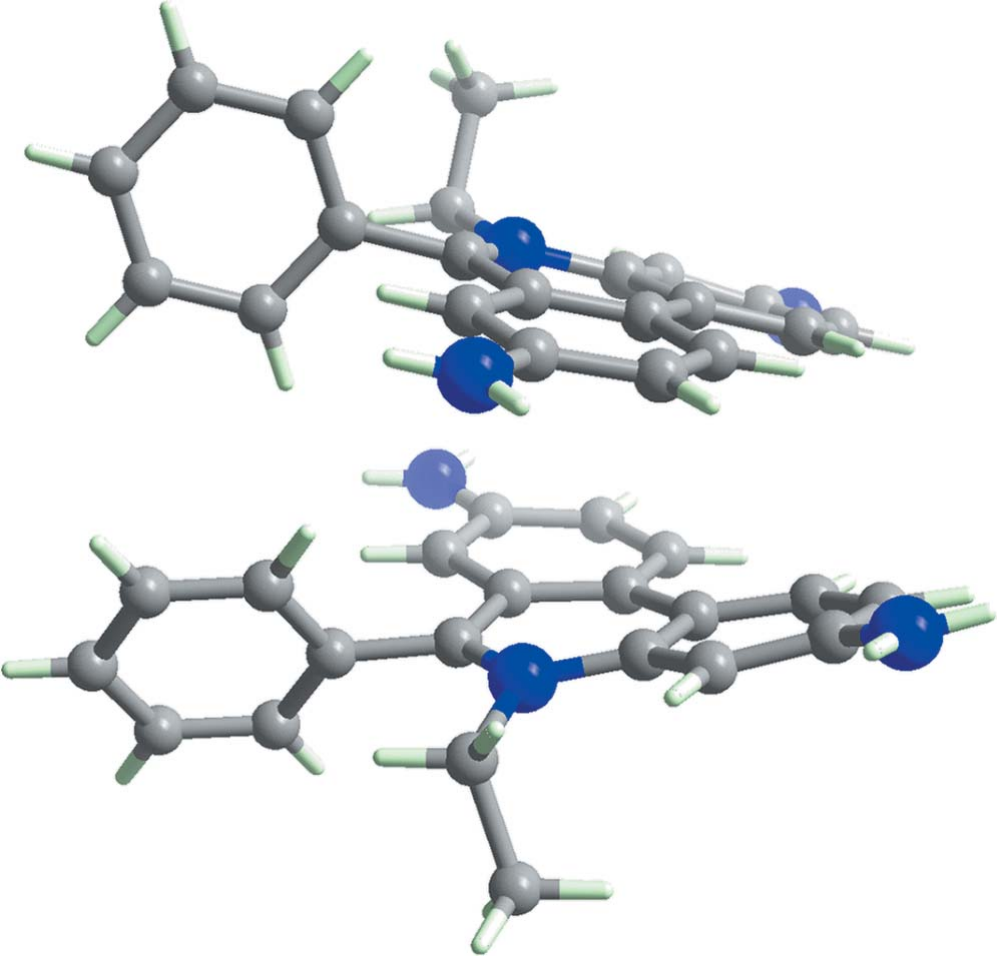
The dimerized structure of the ethidium cations.

**Figure 3:**
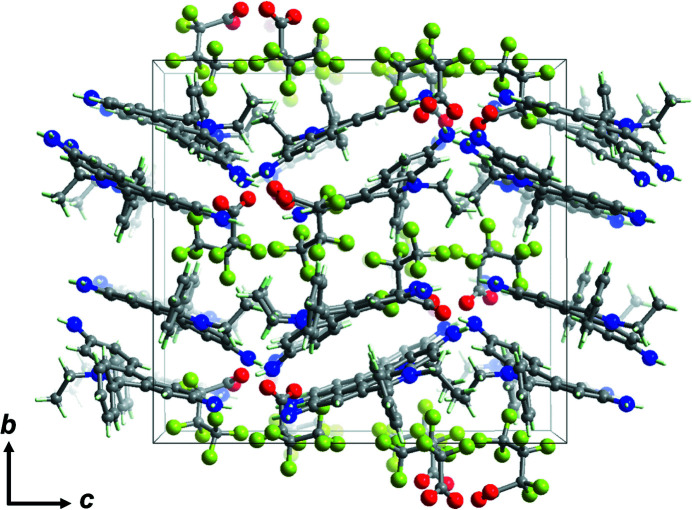
Illustration of the packing of the title compound along the *a* axis.

Ethidium salts are widely used in scientific research as a result of their important applications, including as inter­calators for DNA (Chen *et al.*, 2000 ▸) and as building units for covalent organic frameworks (Ma *et al.*, 2016 ▸). In this study, the structure of an ethidium salt with a hepta­fluoro­butyrate anion is reported (Fig. 1 ▸). Two ethidium cations form a dimerized structure (Fig. 2 ▸) *via* π–π stacking and four dimeric pairs are located in the unit cell. There are two ethidium cations and two hepta­fluoro­butyrate anions as the crystallographically independent components. The ethidium cations do not exhibit a completely planar structure but instead show a slightly bent shape (C19⋯C11⋯C24 = 170.82°, C12⋯C3⋯C25 = 165.57°). The closest *Cg*⋯*Cg* separation between the ethid­ium cations is 3.7502 (3) Å, indicating the presence of a π–π inter­action. Some hydrogen bonds with relatively short distances are observed between the ethidium cation and hepta­fluoro­butyrate anion (*e.g.*, N3—H3*A*⋯O1 = 2.899 Å, N3—H3⋯O4 = 2.909 Å, N5—H5*A*⋯O4 = 2.935 Å, N4—H4*A*⋯O2 = 2.990 Å, N6—H6*A*⋯O3 = 2.939 Å; Table 1 ▸), which would be related to the formation of this packing structure (Fig. 3 ▸).

## Synthesis and crystallization

A methanol solution (1 ml) of silver(I) hepta­fluoro­butyrate (64.2 mg, 0.20 mmol) was mixed with a methanol solution (30 ml) of ethidium bromide (78.9 mg, 0.20 mmol) and then the mixture was stirred for 30 minutes at room temperature. The insoluble precipitate was removed by centrifugation. The remaining solution was evaporated to obtain a crude powder. The crude powder was dissolved in a mixed solvent (methanol:water = 1:1) and red crystals of the target compound were obtained by slow evaporation of the solution after 9 d at room temperature.

## Refinement

Crystal data, data collection and structure refinement details are summarized in Table 2 ▸.

## Supplementary Material

Crystal structure: contains datablock(s) I. DOI: 10.1107/S2414314622008847/xu4048sup1.cif

Structure factors: contains datablock(s) I. DOI: 10.1107/S2414314622008847/xu4048Isup2.hkl

Supporting information file. DOI: 10.1107/S2414314622008847/xu4048Isup3.cdx

CCDC reference: 2205198

Additional supporting information:  crystallographic information; 3D view; checkCIF report

## Figures and Tables

**Table 1:** Hydrogen-bond geometry (Å, °)

*D*—H⋯*A*	*D*—H	H⋯*A*	*D*⋯*A*	*D*—H⋯*A*
N3—H3⋯O4^i^	0.88	2.04	2.909 (4)	171
N3—H3*A*⋯O1^ii^	0.88	2.06	2.899 (3)	159
N4—H4⋯O3^iii^	0.88	2.26	3.088 (4)	158
N4—H4*A*⋯O2	0.88	2.13	2.991 (3)	166
C15—H15⋯O1^iii^	0.95	2.62	3.353 (4)	135
N5—H5*A*⋯O4^iv^	0.88	2.16	2.934 (3)	146
N5—H5*B*⋯O1^iii^	0.88	2.30	3.076 (3)	147
N5—H5*B*⋯F3^iii^	0.88	2.54	3.208 (3)	133
C26—H26⋯F1	0.95	2.61	3.184 (3)	119
N6—H6*A*⋯O3^v^	0.88	2.16	2.938 (3)	147
N6—H6*B*⋯O2^i^	0.88	2.56	3.184 (4)	129
N6—H6*B*⋯F12^i^	0.88	2.34	3.097 (4)	144
C33—H33*A*⋯N3^vi^	0.99	2.59	3.223 (4)	122

Symmetry codes: (i) 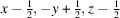 ; (ii) 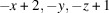 ; (iii) 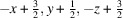 ; (iv) 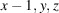 ; (v) 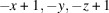 ; (vi) 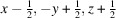 .

**Table 2:** Experimental details

Crystal data	
Chemical formula	C_21_H_20_N_3_^+^·C_4_F_7_O_2_^−^
*M*_r_	527.44
Crystal system, space group	Monoclinic, *P*2_1_/*n*
Temperature (K)	100
*a*, *b*, *c* (Å)	12.1592 (8), 18.9260 (14), 20.3097 (17)
β (°)	91.474 (3)
*V* (Å^3^)	4672.2 (6)
*Z*	8
Radiation type	Mo *K*α
μ (mm^−1^)	0.13
Crystal size (mm)	0.25 × 0.20 × 0.15

Data collection	
Diffractometer	Bruker PHOTON II CPAD
Absorption correction	Multi-scan (*SADABS*; Krause *et al.*, 2015 ▸)
*T*_min_, *T*_max_	0.605, 0.711
No. of measured, independent and observed [*I* > 2σ(*I*)] reflections	58553, 12404, 9416
*R*_int_	0.109
(sin θ/λ)_max_ (Å^−1^)	0.717

Refinement	
*R*[*F* ^2^ > 2σ(*F* ^2^)], *wR*(*F* ^2^), *S*	0.089, 0.218, 1.11
No. of reflections	12404
No. of parameters	669
H-atom treatment	H-atom parameters constrained
Δρ_max_, Δρ_min_ (e Å^−3^)	0.47, −0.45

Computer programs: *APEX4* and *SAINT* (Bruker, 2021 ▸), *SIR2019* (Burla *et al.*, 2015 ▸), *SHELXL2018/3* (Sheldrick, 2015 ▸), *DIAMOND* (Brandenburg, 2014 ▸) and *Yadokari-XG* (Kabuto *et al.*, 2009 ▸).

## full crystallographic data

### Crystal data

**Table:**

C_21_H_20_N_3_^+^·C_4_F_7_O_2_^−^	*F*(000) = 2160
*M**_r_* = 527.44	*D*_x_ = 1.500 Mg m^−^^3^
Monoclinic, *P*2_1_/*n*	Mo *K*α radiation, λ = 0.71069 Å
*a* = 12.1592 (8) Å	Cell parameters from 7105 reflections
*b* = 18.9260 (14) Å	θ = 2.2–27.2°
*c* = 20.3097 (17) Å	µ = 0.13 mm^−^^1^
β = 91.474 (3)°	*T* = 100 K
*V* = 4672.2 (6) Å^3^	Block, red
*Z* = 8	0.25 × 0.20 × 0.15 mm

### Data collection

**Table:**

Bruker PHOTON II CPAD diffractometer	9416 reflections with *I* > 2σ(*I*)
Radiation source: fine-focus sealed tube	*R*_int_ = 0.109
φ and ω scans	θ_max_ = 30.6°, θ_min_ = 1.9°
Absorption correction: multi-scan (SADABS; Krause et al., 2015)	*h* = −16→17
*T*_min_ = 0.605, *T*_max_ = 0.711	*k* = −25→25
58553 measured reflections	*l* = −27→26
12404 independent reflections	

### Refinement

**Table:**

Refinement on *F*^2^	0 restraints
Least-squares matrix: full	Hydrogen site location: inferred from neighbouring sites
*R*[*F*^2^ > 2σ(*F*^2^)] = 0.089	H-atom parameters constrained
*wR*(*F*^2^) = 0.218	*w* = 1/[σ^2^(*F*_o_^2^) + (0.0532*P*)^2^ + 9.0949*P*] where *P* = (*F*_o_^2^ + 2*F*_c_^2^)/3
*S* = 1.11	(Δ/σ)_max_ < 0.001
12404 reflections	Δρ_max_ = 0.47 e Å^−^^3^
669 parameters	Δρ_min_ = −0.45 e Å^−^^3^

### Special details

**Table:**

Geometry. All esds (except the esd in the dihedral angle between two l.s. planes) are estimated using the full covariance matrix. The cell esds are taken into account individually in the estimation of esds in distances, angles and torsion angles; correlations between esds in cell parameters are only used when they are defined by crystal symmetry. An approximate (isotropic) treatment of cell esds is used for estimating esds involving l.s. planes.
Refinement. Refinement of F^2^ against ALL reflections. The weighted R-factor wR and goodness of fit S are based on F^2^, conventional R-factors R are based on F, with F set to zero for negative F^2^. The threshold expression of F^2^ > 2sigma(F^2^) is used only for calculating R-factors(gt) etc. and is not relevant to the choice of reflections for refinement. R-factors based on F^2^ are statistically about twice as large as those based on F, and R- factors based on ALL data will be even larger. All of hydrogen atoms are geometrically fixed using a riding-model approximation with C–H = 0.95 (for phenyl), 0.98 (for methyl), 0.99 (for methylene), and N–H = 0.88 Å.

### Fractional atomic coordinates and isotropic or equivalent isotropic displacement parameters (Å^2^)

**Table:**

	*x*	*y*	*z*	*U*_iso_*/*U*_eq_	
F1	1.10423 (16)	0.34876 (11)	0.57616 (10)	0.0348 (5)	
O1	0.81718 (17)	−0.14092 (12)	0.72561 (11)	0.0272 (5)	
F2	0.69849 (17)	0.00943 (11)	0.65047 (11)	0.0416 (5)	
N1	0.64572 (19)	0.32175 (13)	0.36249 (12)	0.0194 (5)	
N2	0.60689 (19)	0.18575 (12)	0.61439 (12)	0.0190 (5)	
F3	0.96344 (17)	−0.02675 (11)	0.71179 (12)	0.0432 (6)	
F4	1.27314 (17)	0.37587 (12)	0.55497 (10)	0.0396 (5)	
F5	0.91654 (18)	−0.09184 (11)	0.59264 (11)	0.0419 (5)	
F6	1.25336 (18)	0.50412 (11)	0.60348 (12)	0.0447 (6)	
F7	0.8910 (2)	0.07025 (11)	0.67357 (12)	0.0483 (6)	
C1	0.6334 (2)	0.35297 (14)	0.47691 (14)	0.0179 (5)	
F8	0.7581 (2)	−0.00011 (11)	0.75221 (11)	0.0463 (6)	
N3	0.9615 (2)	0.19489 (14)	0.28830 (13)	0.0263 (5)	
H3	0.923744	0.181066	0.253059	0.032*	
H3A	1.029377	0.179993	0.295114	0.032*	
O2	1.15659 (18)	0.33758 (13)	0.70389 (12)	0.0324 (5)	
F9	1.12268 (19)	0.47801 (13)	0.53262 (11)	0.0480 (6)	
O3	0.66684 (18)	−0.12731 (13)	0.66078 (13)	0.0346 (5)	
C2	0.5845 (2)	0.34299 (14)	0.41299 (14)	0.0179 (5)	
F10	0.8540 (2)	0.00707 (14)	0.55507 (12)	0.0572 (7)	
F11	0.98781 (18)	0.45093 (13)	0.64091 (14)	0.0536 (6)	
C3	0.7493 (2)	0.34630 (14)	0.48463 (14)	0.0169 (5)	
C4	0.8107 (2)	0.31722 (14)	0.43182 (14)	0.0176 (5)	
C5	0.7883 (2)	0.13477 (14)	0.44688 (15)	0.0203 (6)	
H5	0.866288	0.137570	0.447140	0.024*	
N4	0.9273 (2)	0.28652 (14)	0.72447 (13)	0.0272 (6)	
H4	0.887391	0.301183	0.757333	0.033*	
H4A	0.997967	0.296386	0.723928	0.033*	
C6	0.5683 (2)	0.37085 (14)	0.53133 (14)	0.0183 (5)	
H6	0.490632	0.374111	0.525647	0.022*	
C7	0.7207 (2)	0.19843 (14)	0.61789 (14)	0.0185 (5)	
C8	0.7560 (2)	0.30125 (14)	0.37144 (14)	0.0179 (5)	
C9	0.5560 (2)	0.16008 (14)	0.56038 (14)	0.0189 (5)	
C10	0.6156 (2)	0.14439 (14)	0.50267 (14)	0.0188 (5)	
C11	0.7837 (2)	0.17952 (14)	0.56289 (14)	0.0181 (5)	
C12	0.6164 (2)	0.38361 (14)	0.59262 (14)	0.0192 (5)	
C13	0.5596 (2)	0.12177 (15)	0.44471 (14)	0.0208 (6)	
H13	0.482034	0.115938	0.444544	0.025*	
C14	0.7315 (2)	0.15223 (14)	0.50495 (14)	0.0178 (5)	
C15	0.7690 (2)	0.23179 (15)	0.67315 (14)	0.0207 (6)	
H15	0.725365	0.242914	0.709883	0.025*	
F12	1.1169 (2)	0.49419 (13)	0.70211 (12)	0.0530 (6)	
C16	0.4646 (2)	0.35300 (15)	0.40168 (14)	0.0194 (5)	
C17	0.8079 (2)	0.26311 (15)	0.32207 (14)	0.0207 (6)	
H17	0.769895	0.253550	0.281587	0.025*	
C18	0.8990 (2)	0.19243 (15)	0.56844 (15)	0.0208 (6)	
H18	0.944662	0.177669	0.533780	0.025*	
C19	0.8796 (2)	0.24854 (15)	0.67443 (15)	0.0215 (6)	
C20	0.9232 (2)	0.29873 (15)	0.43770 (14)	0.0200 (5)	
H20	0.964477	0.312917	0.475781	0.024*	
N5	0.5568 (2)	0.39977 (14)	0.64670 (12)	0.0252 (5)	
H5A	0.484588	0.402420	0.643371	0.030*	
H5B	0.590542	0.407535	0.684857	0.030*	
C21	0.7971 (2)	0.36382 (15)	0.54669 (14)	0.0192 (5)	
H21	0.874886	0.363064	0.552401	0.023*	
C22	0.7334 (2)	0.38185 (15)	0.59871 (14)	0.0202 (6)	
H22	0.768009	0.393437	0.639774	0.024*	
F13	1.0447 (2)	0.55673 (12)	0.62469 (15)	0.0620 (8)	
C23	0.7332 (2)	0.11406 (15)	0.39074 (15)	0.0226 (6)	
H23	0.773730	0.103458	0.352614	0.027*	
C24	0.6170 (2)	0.10799 (15)	0.38796 (14)	0.0212 (6)	
C25	0.9149 (2)	0.23925 (15)	0.33223 (14)	0.0205 (6)	
C26	0.9456 (2)	0.22516 (15)	0.62169 (15)	0.0223 (6)	
H26	1.022906	0.232539	0.623817	0.027*	
N6	0.5651 (2)	0.08560 (15)	0.33075 (13)	0.0296 (6)	
H6A	0.493293	0.079612	0.329379	0.035*	
H6B	0.603644	0.077263	0.295503	0.035*	
C27	0.9738 (2)	0.26090 (16)	0.38982 (15)	0.0218 (6)	
H27	1.049400	0.248987	0.395220	0.026*	
C28	0.4343 (2)	0.14853 (15)	0.56064 (14)	0.0205 (6)	
O4	1.3299 (2)	0.36498 (19)	0.68042 (15)	0.0545 (9)	
C29	1.2309 (2)	0.35991 (17)	0.66902 (16)	0.0260 (6)	
C30	0.4161 (2)	0.41967 (16)	0.40651 (15)	0.0239 (6)	
H30	0.460104	0.460049	0.415984	0.029*	
C31	0.3991 (2)	0.29412 (16)	0.38733 (15)	0.0230 (6)	
H31	0.431873	0.248751	0.383922	0.028*	
C32	0.3920 (3)	0.08359 (16)	0.57879 (18)	0.0290 (7)	
H32	0.440069	0.046024	0.591124	0.035*	
C33	0.5421 (2)	0.19980 (16)	0.67456 (14)	0.0223 (6)	
H33	0.463424	0.205209	0.661902	0.027*	
H33A	0.567472	0.244567	0.695105	0.027*	
C34	0.2383 (2)	0.36769 (18)	0.38333 (16)	0.0278 (7)	
H34	0.160998	0.372794	0.377353	0.033*	
C35	0.7493 (2)	−0.10657 (17)	0.69107 (15)	0.0244 (6)	
C36	0.2866 (2)	0.30157 (17)	0.37806 (16)	0.0266 (6)	
H36	0.242373	0.261402	0.368075	0.032*	
C37	0.6013 (2)	0.32601 (18)	0.29317 (15)	0.0259 (6)	
H37	0.520477	0.332442	0.293546	0.031*	
H37A	0.616777	0.281270	0.269924	0.031*	
C38	0.8860 (3)	−0.00120 (17)	0.66979 (17)	0.0303 (7)	
C39	0.3034 (3)	0.42642 (17)	0.39738 (16)	0.0284 (7)	
H39	0.270132	0.471666	0.400746	0.034*	
C40	0.2508 (3)	0.19222 (18)	0.54011 (17)	0.0299 (7)	
H40	0.202339	0.229124	0.526610	0.036*	
C41	1.1930 (2)	0.38599 (17)	0.59907 (16)	0.0262 (6)	
C42	0.5551 (3)	0.14010 (18)	0.72407 (16)	0.0303 (7)	
H42	0.525777	0.096331	0.704727	0.046*	
H42A	0.514629	0.151654	0.763772	0.046*	
H42B	0.633197	0.133786	0.735664	0.046*	
C43	1.1618 (3)	0.46488 (18)	0.59370 (17)	0.0303 (7)	
C44	0.3635 (2)	0.20300 (16)	0.54076 (16)	0.0254 (6)	
H44	0.392532	0.247307	0.527726	0.030*	
C45	0.7712 (3)	−0.02542 (17)	0.69042 (16)	0.0270 (6)	
F14	1.0221 (2)	−0.00067 (16)	0.59017 (17)	0.0744 (9)	
C46	0.2090 (3)	0.12757 (19)	0.55915 (19)	0.0349 (8)	
H46	0.131748	0.120142	0.558691	0.042*	
C47	0.2793 (3)	0.07386 (18)	0.5788 (2)	0.0378 (8)	
H47	0.249992	0.029861	0.592485	0.045*	
C48	0.6529 (3)	0.3871 (2)	0.25683 (17)	0.0380 (8)	
H48	0.636602	0.431470	0.279466	0.057*	
H48A	0.622816	0.388958	0.211641	0.057*	
H48B	0.732801	0.380377	0.255904	0.057*	
C49	1.0752 (3)	0.49156 (19)	0.6410 (2)	0.0380 (8)	
C50	0.9189 (3)	−0.0216 (2)	0.6003 (2)	0.0398 (9)	

### Atomic displacement parameters (Å^2^)

**Table:**

	*U*^11^	*U*^22^	*U*^33^	*U*^12^	*U*^13^	*U*^23^
F1	0.0375 (11)	0.0321 (10)	0.0343 (11)	−0.0072 (8)	−0.0076 (8)	−0.0019 (8)
O1	0.0229 (10)	0.0265 (11)	0.0321 (12)	−0.0015 (8)	0.0014 (9)	0.0064 (9)
F2	0.0363 (11)	0.0372 (12)	0.0511 (13)	0.0142 (9)	−0.0055 (9)	0.0075 (10)
N1	0.0186 (11)	0.0224 (12)	0.0172 (12)	−0.0015 (9)	0.0004 (9)	−0.0017 (9)
N2	0.0182 (11)	0.0184 (11)	0.0208 (12)	0.0014 (9)	0.0052 (9)	−0.0012 (9)
F3	0.0340 (11)	0.0366 (12)	0.0578 (14)	−0.0118 (9)	−0.0205 (10)	0.0162 (10)
F4	0.0418 (11)	0.0466 (12)	0.0312 (11)	0.0077 (9)	0.0151 (9)	0.0037 (9)
F5	0.0514 (13)	0.0348 (11)	0.0401 (12)	0.0074 (9)	0.0159 (10)	0.0062 (9)
F6	0.0427 (12)	0.0328 (11)	0.0590 (15)	−0.0151 (9)	0.0084 (10)	0.0105 (10)
F7	0.0676 (15)	0.0212 (10)	0.0555 (15)	−0.0098 (10)	−0.0093 (12)	0.0071 (9)
C1	0.0197 (13)	0.0133 (12)	0.0207 (14)	−0.0029 (9)	0.0005 (10)	0.0000 (10)
F8	0.0762 (16)	0.0325 (11)	0.0308 (11)	0.0037 (10)	0.0115 (10)	−0.0092 (9)
N3	0.0231 (12)	0.0319 (14)	0.0239 (13)	0.0063 (10)	0.0040 (10)	−0.0028 (11)
O2	0.0248 (11)	0.0415 (14)	0.0310 (12)	−0.0027 (10)	0.0021 (9)	0.0082 (10)
F9	0.0575 (14)	0.0498 (14)	0.0365 (12)	0.0109 (11)	−0.0017 (10)	0.0192 (10)
O3	0.0241 (11)	0.0366 (13)	0.0427 (14)	−0.0017 (9)	−0.0050 (10)	−0.0055 (11)
C2	0.0181 (12)	0.0151 (12)	0.0204 (14)	−0.0012 (10)	0.0007 (10)	0.0008 (10)
F10	0.0867 (19)	0.0517 (15)	0.0334 (12)	0.0229 (13)	0.0070 (12)	0.0139 (11)
F11	0.0338 (11)	0.0446 (14)	0.0832 (19)	−0.0017 (10)	0.0185 (11)	0.0006 (12)
C3	0.0190 (12)	0.0128 (12)	0.0189 (13)	−0.0022 (9)	0.0002 (10)	0.0020 (10)
C4	0.0179 (12)	0.0145 (12)	0.0205 (14)	−0.0038 (9)	−0.0004 (10)	0.0021 (10)
C5	0.0194 (13)	0.0152 (13)	0.0265 (15)	−0.0003 (10)	0.0046 (11)	0.0019 (11)
N4	0.0213 (12)	0.0302 (14)	0.0297 (14)	0.0004 (10)	−0.0042 (10)	−0.0048 (11)
C6	0.0169 (12)	0.0176 (13)	0.0203 (14)	−0.0014 (10)	0.0001 (10)	0.0003 (10)
C7	0.0170 (12)	0.0172 (13)	0.0212 (14)	0.0006 (10)	0.0018 (10)	0.0036 (10)
C8	0.0173 (12)	0.0178 (13)	0.0185 (13)	−0.0012 (10)	0.0014 (10)	0.0012 (10)
C9	0.0190 (13)	0.0160 (13)	0.0218 (14)	0.0017 (10)	0.0020 (10)	0.0026 (10)
C10	0.0218 (13)	0.0150 (12)	0.0196 (14)	0.0014 (10)	0.0033 (10)	0.0028 (10)
C11	0.0204 (13)	0.0150 (12)	0.0192 (13)	0.0021 (10)	0.0040 (10)	0.0039 (10)
C12	0.0217 (13)	0.0150 (12)	0.0211 (14)	−0.0017 (10)	0.0025 (11)	0.0013 (10)
C13	0.0190 (13)	0.0205 (14)	0.0229 (14)	0.0020 (10)	0.0010 (11)	0.0026 (11)
C14	0.0189 (12)	0.0145 (12)	0.0202 (14)	0.0016 (9)	0.0046 (10)	0.0041 (10)
C15	0.0215 (13)	0.0199 (13)	0.0208 (14)	0.0033 (10)	0.0029 (11)	0.0016 (11)
F12	0.0798 (18)	0.0401 (13)	0.0397 (13)	−0.0022 (12)	0.0134 (12)	−0.0063 (10)
C16	0.0185 (13)	0.0216 (14)	0.0180 (13)	−0.0005 (10)	0.0001 (10)	0.0002 (10)
C17	0.0202 (13)	0.0231 (14)	0.0188 (14)	−0.0022 (11)	0.0016 (10)	0.0000 (11)
C18	0.0177 (13)	0.0196 (13)	0.0254 (15)	0.0031 (10)	0.0051 (11)	0.0037 (11)
C19	0.0243 (14)	0.0172 (13)	0.0230 (14)	0.0008 (10)	−0.0021 (11)	0.0023 (11)
C20	0.0167 (12)	0.0210 (13)	0.0223 (14)	−0.0028 (10)	0.0008 (10)	0.0022 (11)
N5	0.0215 (12)	0.0357 (15)	0.0184 (12)	−0.0032 (10)	0.0023 (9)	−0.0032 (10)
C21	0.0189 (13)	0.0186 (13)	0.0199 (14)	0.0004 (10)	−0.0029 (10)	0.0020 (10)
C22	0.0227 (13)	0.0172 (13)	0.0204 (14)	−0.0020 (10)	−0.0022 (11)	−0.0003 (10)
F13	0.0771 (18)	0.0307 (12)	0.0795 (19)	0.0179 (11)	0.0275 (15)	0.0170 (12)
C23	0.0274 (14)	0.0182 (13)	0.0225 (15)	0.0031 (11)	0.0100 (11)	0.0024 (11)
C24	0.0254 (14)	0.0169 (13)	0.0211 (14)	0.0037 (10)	0.0008 (11)	0.0021 (11)
C25	0.0202 (13)	0.0204 (13)	0.0212 (14)	−0.0032 (10)	0.0060 (11)	0.0017 (11)
C26	0.0184 (13)	0.0193 (14)	0.0293 (16)	−0.0004 (10)	0.0024 (11)	0.0052 (11)
N6	0.0261 (13)	0.0400 (16)	0.0225 (13)	0.0026 (11)	0.0012 (10)	−0.0031 (11)
C27	0.0162 (12)	0.0235 (14)	0.0259 (15)	−0.0008 (10)	0.0021 (11)	0.0051 (11)
C28	0.0218 (13)	0.0202 (13)	0.0199 (14)	−0.0012 (10)	0.0047 (11)	−0.0007 (11)
O4	0.0221 (12)	0.093 (2)	0.0479 (17)	−0.0125 (13)	−0.0055 (11)	0.0342 (16)
C29	0.0220 (14)	0.0268 (15)	0.0293 (16)	−0.0008 (11)	−0.0006 (12)	0.0077 (12)
C30	0.0271 (15)	0.0204 (14)	0.0240 (15)	0.0000 (11)	−0.0030 (12)	0.0018 (11)
C31	0.0214 (13)	0.0216 (14)	0.0259 (15)	−0.0011 (11)	0.0003 (11)	−0.0020 (11)
C32	0.0234 (15)	0.0194 (14)	0.045 (2)	0.0017 (11)	0.0037 (13)	0.0027 (13)
C33	0.0196 (13)	0.0270 (15)	0.0207 (14)	0.0022 (11)	0.0077 (11)	−0.0030 (11)
C34	0.0168 (13)	0.0363 (17)	0.0302 (17)	0.0034 (12)	−0.0005 (12)	0.0008 (13)
C35	0.0202 (14)	0.0287 (16)	0.0244 (15)	0.0024 (11)	0.0045 (11)	0.0002 (12)
C36	0.0193 (14)	0.0304 (16)	0.0300 (16)	−0.0032 (12)	0.0007 (12)	−0.0028 (13)
C37	0.0199 (13)	0.0395 (18)	0.0182 (14)	0.0009 (12)	−0.0030 (11)	−0.0041 (12)
C38	0.0333 (17)	0.0211 (15)	0.0361 (18)	−0.0038 (12)	−0.0069 (14)	0.0067 (13)
C39	0.0305 (16)	0.0220 (15)	0.0324 (17)	0.0082 (12)	−0.0027 (13)	0.0024 (12)
C40	0.0223 (14)	0.0293 (16)	0.0381 (18)	0.0070 (12)	0.0024 (13)	0.0008 (14)
C41	0.0236 (14)	0.0300 (16)	0.0252 (16)	−0.0015 (12)	0.0030 (12)	0.0020 (12)
C42	0.0347 (17)	0.0319 (17)	0.0249 (16)	0.0002 (13)	0.0108 (13)	−0.0008 (13)
C43	0.0310 (16)	0.0307 (17)	0.0292 (17)	−0.0023 (13)	0.0011 (13)	0.0073 (13)
C44	0.0230 (14)	0.0221 (14)	0.0312 (16)	0.0007 (11)	0.0031 (12)	0.0042 (12)
C45	0.0307 (16)	0.0248 (15)	0.0254 (16)	0.0078 (12)	−0.0009 (12)	−0.0005 (12)
F14	0.0552 (16)	0.0700 (19)	0.100 (2)	−0.0157 (14)	0.0377 (16)	0.0174 (17)
C46	0.0200 (14)	0.0347 (18)	0.050 (2)	−0.0007 (13)	0.0045 (14)	−0.0023 (16)
C47	0.0261 (16)	0.0257 (17)	0.062 (2)	−0.0063 (13)	0.0062 (16)	0.0044 (16)
C48	0.0379 (19)	0.051 (2)	0.0247 (17)	0.0022 (16)	−0.0039 (14)	0.0114 (15)
C49	0.043 (2)	0.0252 (17)	0.047 (2)	0.0019 (14)	0.0104 (16)	0.0055 (15)
C50	0.0394 (19)	0.036 (2)	0.045 (2)	0.0013 (15)	0.0103 (16)	0.0153 (16)

### Geometric parameters (Å, º)

**Table:**

F1—C41	1.361 (4)	C18—C26	1.358 (4)
O1—C35	1.252 (4)	C18—H18	0.9500
F2—C45	1.356 (4)	C19—C26	1.426 (4)
N1—C2	1.344 (4)	C20—C27	1.367 (4)
N1—C8	1.403 (3)	C20—H20	0.9500
N1—C37	1.497 (4)	N5—H5A	0.8800
N2—C9	1.337 (4)	N5—H5B	0.8800
N2—C7	1.405 (3)	C21—C22	1.369 (4)
N2—C33	1.494 (3)	C21—H21	0.9500
F3—C38	1.344 (4)	C22—H22	0.9500
F4—C41	1.354 (4)	F13—C49	1.328 (4)
F5—C50	1.338 (4)	C23—C24	1.417 (4)
F6—C43	1.349 (4)	C23—H23	0.9500
F7—C38	1.356 (4)	C24—N6	1.375 (4)
C1—C6	1.417 (4)	C25—C27	1.416 (4)
C1—C3	1.419 (4)	C26—H26	0.9500
C1—C2	1.427 (4)	N6—H6A	0.8800
F8—C45	1.356 (4)	N6—H6B	0.8800
N3—C25	1.359 (4)	C27—H27	0.9500
N3—H3	0.8800	C28—C32	1.386 (4)
N3—H3A	0.8800	C28—C44	1.396 (4)
O2—C29	1.237 (4)	O4—C29	1.223 (4)
F9—C43	1.340 (4)	C29—C41	1.562 (4)
O3—C35	1.227 (4)	C30—C39	1.384 (4)
C2—C16	1.482 (4)	C30—H30	0.9500
F10—C50	1.313 (4)	C31—C36	1.383 (4)
F11—C49	1.312 (4)	C31—H31	0.9500
C3—C21	1.414 (4)	C32—C47	1.382 (4)
C3—C4	1.432 (4)	C32—H32	0.9500
C4—C8	1.413 (4)	C33—C42	1.518 (4)
C4—C20	1.414 (4)	C33—H33	0.9900
C5—C23	1.365 (4)	C33—H33A	0.9900
C5—C14	1.421 (4)	C34—C36	1.388 (5)
C5—H5	0.9500	C34—C39	1.389 (5)
N4—C19	1.362 (4)	C34—H34	0.9500
N4—H4	0.8800	C35—C45	1.559 (4)
N4—H4A	0.8800	C36—H36	0.9500
C6—C12	1.383 (4)	C37—C48	1.517 (5)
C6—H6	0.9500	C37—H37	0.9900
C7—C15	1.403 (4)	C37—H37A	0.9900
C7—C11	1.416 (4)	C38—C50	1.526 (5)
C8—C17	1.399 (4)	C38—C45	1.538 (5)
C9—C10	1.425 (4)	C39—H39	0.9500
C9—C28	1.496 (4)	C40—C46	1.384 (5)
C10—C13	1.411 (4)	C40—C44	1.385 (4)
C10—C14	1.416 (4)	C40—H40	0.9500
C11—C14	1.420 (4)	C41—C43	1.544 (5)
C11—C18	1.424 (4)	C42—H42	0.9800
C12—N5	1.366 (4)	C42—H42A	0.9800
C12—C22	1.425 (4)	C42—H42B	0.9800
C13—C24	1.387 (4)	C43—C49	1.529 (5)
C13—H13	0.9500	C44—H44	0.9500
C15—C19	1.381 (4)	F14—C50	1.337 (4)
C15—H15	0.9500	C46—C47	1.380 (5)
F12—C49	1.330 (5)	C46—H46	0.9500
C16—C31	1.397 (4)	C47—H47	0.9500
C16—C30	1.397 (4)	C48—H48	0.9800
C17—C25	1.387 (4)	C48—H48A	0.9800
C17—H17	0.9500	C48—H48B	0.9800
			
C2—N1—C8	122.0 (2)	C32—C28—C44	120.1 (3)
C2—N1—C37	120.5 (2)	C32—C28—C9	120.3 (3)
C8—N1—C37	117.2 (2)	C44—C28—C9	119.6 (3)
C9—N2—C7	122.5 (2)	O4—C29—O2	130.4 (3)
C9—N2—C33	119.6 (2)	O4—C29—C41	114.4 (3)
C7—N2—C33	117.9 (2)	O2—C29—C41	115.2 (3)
C6—C1—C3	120.5 (3)	C39—C30—C16	119.5 (3)
C6—C1—C2	120.9 (2)	C39—C30—H30	120.2
C3—C1—C2	118.6 (2)	C16—C30—H30	120.2
C25—N3—H3	120.0	C36—C31—C16	120.3 (3)
C25—N3—H3A	120.0	C36—C31—H31	119.9
H3—N3—H3A	120.0	C16—C31—H31	119.9
N1—C2—C1	120.5 (2)	C47—C32—C28	119.5 (3)
N1—C2—C16	118.8 (2)	C47—C32—H32	120.2
C1—C2—C16	120.6 (2)	C28—C32—H32	120.2
C21—C3—C1	117.5 (2)	N2—C33—C42	111.2 (2)
C21—C3—C4	123.2 (2)	N2—C33—H33	109.4
C1—C3—C4	119.1 (2)	C42—C33—H33	109.4
C8—C4—C20	117.0 (3)	N2—C33—H33A	109.4
C8—C4—C3	119.3 (2)	C42—C33—H33A	109.4
C20—C4—C3	123.6 (3)	H33—C33—H33A	108.0
C23—C5—C14	121.4 (3)	C36—C34—C39	119.9 (3)
C23—C5—H5	119.3	C36—C34—H34	120.1
C14—C5—H5	119.3	C39—C34—H34	120.1
C19—N4—H4	120.0	O3—C35—O1	129.3 (3)
C19—N4—H4A	120.0	O3—C35—C45	116.7 (3)
H4—N4—H4A	120.0	O1—C35—C45	113.9 (3)
C12—C6—C1	120.8 (2)	C31—C36—C34	120.0 (3)
C12—C6—H6	119.6	C31—C36—H36	120.0
C1—C6—H6	119.6	C34—C36—H36	120.0
C15—C7—N2	120.6 (2)	N1—C37—C48	110.8 (3)
C15—C7—C11	121.4 (2)	N1—C37—H37	109.5
N2—C7—C11	118.0 (3)	C48—C37—H37	109.5
C17—C8—N1	119.8 (2)	N1—C37—H37A	109.5
C17—C8—C4	121.4 (2)	C48—C37—H37A	109.5
N1—C8—C4	118.7 (2)	H37—C37—H37A	108.1
N2—C9—C10	121.1 (2)	F3—C38—F7	107.0 (3)
N2—C9—C28	119.1 (2)	F3—C38—C50	107.4 (3)
C10—C9—C28	119.7 (3)	F7—C38—C50	107.0 (3)
C13—C10—C14	121.1 (2)	F3—C38—C45	110.2 (3)
C13—C10—C9	120.3 (2)	F7—C38—C45	108.8 (3)
C14—C10—C9	118.5 (3)	C50—C38—C45	116.0 (3)
C7—C11—C14	120.4 (2)	C30—C39—C34	120.7 (3)
C7—C11—C18	116.4 (3)	C30—C39—H39	119.7
C14—C11—C18	123.1 (3)	C34—C39—H39	119.7
N5—C12—C6	122.8 (3)	C46—C40—C44	119.9 (3)
N5—C12—C22	119.0 (3)	C46—C40—H40	120.1
C6—C12—C22	118.2 (3)	C44—C40—H40	120.1
C24—C13—C10	120.5 (3)	F4—C41—F1	106.2 (3)
C24—C13—H13	119.7	F4—C41—C43	105.7 (3)
C10—C13—H13	119.7	F1—C41—C43	106.5 (2)
C10—C14—C11	119.2 (2)	F4—C41—C29	110.9 (2)
C10—C14—C5	116.9 (3)	F1—C41—C29	111.1 (2)
C11—C14—C5	123.8 (2)	C43—C41—C29	115.9 (3)
C19—C15—C7	120.3 (3)	C33—C42—H42	109.5
C19—C15—H15	119.8	C33—C42—H42A	109.5
C7—C15—H15	119.8	H42—C42—H42A	109.5
C31—C16—C30	119.7 (3)	C33—C42—H42B	109.5
C31—C16—C2	119.0 (3)	H42—C42—H42B	109.5
C30—C16—C2	121.3 (3)	H42A—C42—H42B	109.5
C25—C17—C8	120.0 (3)	F9—C43—F6	107.8 (3)
C25—C17—H17	120.0	F9—C43—C49	106.7 (3)
C8—C17—H17	120.0	F6—C43—C49	107.7 (3)
C26—C18—C11	122.1 (3)	F9—C43—C41	109.0 (3)
C26—C18—H18	118.9	F6—C43—C41	108.7 (3)
C11—C18—H18	118.9	C49—C43—C41	116.6 (3)
N4—C19—C15	122.1 (3)	C40—C44—C28	119.8 (3)
N4—C19—C26	119.2 (3)	C40—C44—H44	120.1
C15—C19—C26	118.8 (3)	C28—C44—H44	120.1
C27—C20—C4	121.5 (3)	F2—C45—F8	107.0 (3)
C27—C20—H20	119.3	F2—C45—C38	105.9 (3)
C4—C20—H20	119.3	F8—C45—C38	106.0 (3)
C12—N5—H5A	120.0	F2—C45—C35	112.1 (3)
C12—N5—H5B	120.0	F8—C45—C35	108.4 (3)
H5A—N5—H5B	120.0	C38—C45—C35	116.9 (2)
C22—C21—C3	121.2 (3)	C47—C46—C40	120.2 (3)
C22—C21—H21	119.4	C47—C46—H46	119.9
C3—C21—H21	119.4	C40—C46—H46	119.9
C21—C22—C12	121.5 (3)	C46—C47—C32	120.5 (3)
C21—C22—H22	119.3	C46—C47—H47	119.7
C12—C22—H22	119.3	C32—C47—H47	119.7
C5—C23—C24	121.6 (3)	C37—C48—H48	109.5
C5—C23—H23	119.2	C37—C48—H48A	109.5
C24—C23—H23	119.2	H48—C48—H48A	109.5
N6—C24—C13	122.0 (3)	C37—C48—H48B	109.5
N6—C24—C23	119.6 (3)	H48—C48—H48B	109.5
C13—C24—C23	118.3 (3)	H48A—C48—H48B	109.5
N3—C25—C17	120.6 (3)	F11—C49—F13	108.8 (3)
N3—C25—C27	120.7 (3)	F11—C49—F12	108.2 (3)
C17—C25—C27	118.8 (3)	F13—C49—F12	107.2 (3)
C18—C26—C19	120.5 (3)	F11—C49—C43	112.1 (3)
C18—C26—H26	119.7	F13—C49—C43	110.1 (3)
C19—C26—H26	119.7	F12—C49—C43	110.3 (3)
C24—N6—H6A	120.0	F10—C50—F14	108.6 (3)
C24—N6—H6B	120.0	F10—C50—F5	108.6 (3)
H6A—N6—H6B	120.0	F14—C50—F5	107.1 (3)
C20—C27—C25	120.8 (3)	F10—C50—C38	112.0 (3)
C20—C27—H27	119.6	F14—C50—C38	109.7 (3)
C25—C27—H27	119.6	F5—C50—C38	110.7 (3)
			
C8—N1—C2—C1	7.0 (4)	C8—C17—C25—N3	171.8 (3)
C37—N1—C2—C1	−166.9 (3)	C8—C17—C25—C27	−7.0 (4)
C8—N1—C2—C16	−170.5 (2)	C11—C18—C26—C19	−0.5 (4)
C37—N1—C2—C16	15.6 (4)	N4—C19—C26—C18	−174.4 (3)
C6—C1—C2—N1	−175.8 (3)	C15—C19—C26—C18	5.7 (4)
C3—C1—C2—N1	5.7 (4)	C4—C20—C27—C25	0.4 (4)
C6—C1—C2—C16	1.7 (4)	N3—C25—C27—C20	−172.4 (3)
C3—C1—C2—C16	−176.8 (2)	C17—C25—C27—C20	6.4 (4)
C6—C1—C3—C21	−5.1 (4)	N2—C9—C28—C32	−91.9 (4)
C2—C1—C3—C21	173.4 (2)	C10—C9—C28—C32	88.8 (4)
C6—C1—C3—C4	170.0 (2)	N2—C9—C28—C44	89.8 (3)
C2—C1—C3—C4	−11.5 (4)	C10—C9—C28—C44	−89.5 (3)
C21—C3—C4—C8	180.0 (3)	C31—C16—C30—C39	0.4 (4)
C1—C3—C4—C8	5.1 (4)	C2—C16—C30—C39	−178.2 (3)
C21—C3—C4—C20	4.4 (4)	C30—C16—C31—C36	−0.1 (4)
C1—C3—C4—C20	−170.4 (3)	C2—C16—C31—C36	178.5 (3)
C3—C1—C6—C12	1.4 (4)	C44—C28—C32—C47	−1.8 (5)
C2—C1—C6—C12	−177.1 (3)	C9—C28—C32—C47	179.9 (3)
C9—N2—C7—C15	173.7 (3)	C9—N2—C33—C42	99.4 (3)
C33—N2—C7—C15	−8.1 (4)	C7—N2—C33—C42	−78.8 (3)
C9—N2—C7—C11	−3.4 (4)	C16—C31—C36—C34	−0.3 (5)
C33—N2—C7—C11	174.7 (2)	C39—C34—C36—C31	0.6 (5)
C2—N1—C8—C17	163.8 (3)	C2—N1—C37—C48	105.0 (3)
C37—N1—C8—C17	−22.1 (4)	C8—N1—C37—C48	−69.2 (3)
C2—N1—C8—C4	−13.5 (4)	C16—C30—C39—C34	−0.2 (5)
C37—N1—C8—C4	160.6 (3)	C36—C34—C39—C30	−0.3 (5)
C20—C4—C8—C17	5.7 (4)	O4—C29—C41—F4	30.7 (4)
C3—C4—C8—C17	−170.1 (3)	O2—C29—C41—F4	−149.4 (3)
C20—C4—C8—N1	−177.0 (2)	O4—C29—C41—F1	148.5 (3)
C3—C4—C8—N1	7.1 (4)	O2—C29—C41—F1	−31.6 (4)
C7—N2—C9—C10	−0.3 (4)	O4—C29—C41—C43	−89.8 (4)
C33—N2—C9—C10	−178.4 (2)	O2—C29—C41—C43	90.2 (4)
C7—N2—C9—C28	−179.6 (2)	F4—C41—C43—F9	61.8 (3)
C33—N2—C9—C28	2.3 (4)	F1—C41—C43—F9	−50.9 (3)
N2—C9—C10—C13	−175.8 (3)	C29—C41—C43—F9	−175.0 (2)
C28—C9—C10—C13	3.5 (4)	F4—C41—C43—F6	−55.5 (3)
N2—C9—C10—C14	4.0 (4)	F1—C41—C43—F6	−168.1 (2)
C28—C9—C10—C14	−176.7 (2)	C29—C41—C43—F6	67.7 (3)
C15—C7—C11—C14	−173.7 (3)	F4—C41—C43—C49	−177.4 (3)
N2—C7—C11—C14	3.4 (4)	F1—C41—C43—C49	69.9 (4)
C15—C7—C11—C18	3.5 (4)	C29—C41—C43—C49	−54.2 (4)
N2—C7—C11—C18	−179.4 (2)	C46—C40—C44—C28	0.0 (5)
C1—C6—C12—N5	−178.8 (3)	C32—C28—C44—C40	0.9 (5)
C1—C6—C12—C22	3.2 (4)	C9—C28—C44—C40	179.2 (3)
C14—C10—C13—C24	−1.3 (4)	F3—C38—C45—F2	172.9 (3)
C9—C10—C13—C24	178.5 (3)	F7—C38—C45—F2	55.8 (3)
C13—C10—C14—C11	175.9 (3)	C50—C38—C45—F2	−64.8 (3)
C9—C10—C14—C11	−3.9 (4)	F3—C38—C45—F8	59.4 (3)
C13—C10—C14—C5	−1.4 (4)	F7—C38—C45—F8	−57.6 (3)
C9—C10—C14—C5	178.8 (2)	C50—C38—C45—F8	−178.2 (3)
C7—C11—C14—C10	0.3 (4)	F3—C38—C45—C35	−61.5 (4)
C18—C11—C14—C10	−176.8 (2)	F7—C38—C45—C35	−178.6 (3)
C7—C11—C14—C5	177.4 (3)	C50—C38—C45—C35	60.8 (4)
C18—C11—C14—C5	0.3 (4)	O3—C35—C45—F2	−5.6 (4)
C23—C5—C14—C10	2.5 (4)	O1—C35—C45—F2	177.6 (3)
C23—C5—C14—C11	−174.7 (3)	O3—C35—C45—F8	112.3 (3)
N2—C7—C15—C19	−175.5 (3)	O1—C35—C45—F8	−64.6 (3)
C11—C7—C15—C19	1.5 (4)	O3—C35—C45—C38	−128.1 (3)
N1—C2—C16—C31	66.4 (4)	O1—C35—C45—C38	55.1 (4)
C1—C2—C16—C31	−111.1 (3)	C44—C40—C46—C47	0.0 (6)
N1—C2—C16—C30	−115.0 (3)	C40—C46—C47—C32	−1.0 (6)
C1—C2—C16—C30	67.5 (4)	C28—C32—C47—C46	1.9 (6)
N1—C8—C17—C25	−176.3 (3)	F9—C43—C49—F11	72.4 (4)
C4—C8—C17—C25	0.9 (4)	F6—C43—C49—F11	−172.1 (3)
C7—C11—C18—C26	−4.1 (4)	C41—C43—C49—F11	−49.6 (4)
C14—C11—C18—C26	173.2 (3)	F9—C43—C49—F13	−48.9 (4)
C7—C15—C19—N4	174.0 (3)	F6—C43—C49—F13	66.7 (4)
C7—C15—C19—C26	−6.1 (4)	C41—C43—C49—F13	−170.9 (3)
C8—C4—C20—C27	−6.3 (4)	F9—C43—C49—F12	−166.9 (3)
C3—C4—C20—C27	169.3 (3)	F6—C43—C49—F12	−51.4 (4)
C1—C3—C21—C22	4.4 (4)	C41—C43—C49—F12	71.1 (4)
C4—C3—C21—C22	−170.5 (3)	F3—C38—C50—F10	−173.6 (3)
C3—C21—C22—C12	0.1 (4)	F7—C38—C50—F10	−59.0 (4)
N5—C12—C22—C21	177.9 (3)	C45—C38—C50—F10	62.6 (4)
C6—C12—C22—C21	−4.0 (4)	F3—C38—C50—F14	−52.9 (4)
C14—C5—C23—C24	−0.9 (4)	F7—C38—C50—F14	61.7 (4)
C10—C13—C24—N6	179.7 (3)	C45—C38—C50—F14	−176.7 (3)
C10—C13—C24—C23	2.9 (4)	F3—C38—C50—F5	65.1 (4)
C5—C23—C24—N6	−178.7 (3)	F7—C38—C50—F5	179.7 (3)
C5—C23—C24—C13	−1.8 (4)	C45—C38—C50—F5	−58.8 (4)

### Hydrogen-bond geometry (Å, º)

**Table:**

*D*—H···*A*	*D*—H	H···*A*	*D*···*A*	*D*—H···*A*
N3—H3···O4^i^	0.88	2.04	2.909 (4)	171
N3—H3*A*···O1^ii^	0.88	2.06	2.899 (3)	159
N4—H4···O3^iii^	0.88	2.26	3.088 (4)	158
N4—H4*A*···O2	0.88	2.13	2.991 (3)	166
C15—H15···O1^iii^	0.95	2.62	3.353 (4)	135
N5—H5*A*···O4^iv^	0.88	2.16	2.934 (3)	146
N5—H5*B*···O1^iii^	0.88	2.30	3.076 (3)	147
N5—H5*B*···F3^iii^	0.88	2.54	3.208 (3)	133
C26—H26···F1	0.95	2.61	3.184 (3)	119
N6—H6*A*···O3^v^	0.88	2.16	2.938 (3)	147
N6—H6*B*···O2^i^	0.88	2.56	3.184 (4)	129
N6—H6*B*···F12^i^	0.88	2.34	3.097 (4)	144
C33—H33*A*···N3^vi^	0.99	2.59	3.223 (4)	122

Symmetry codes: (i) *x*−1/2, −*y*+1/2, *z*−1/2; (ii) −*x*+2, −*y*, −*z*+1; (iii) −*x*+3/2, *y*+1/2, −*z*+3/2; (iv) *x*−1, *y*, *z*; (v) −*x*+1, −*y*, −*z*+1; (vi) *x*−1/2, −*y*+1/2, *z*+1/2.
